# Paging Doctor Google! Heuristics vs. technology

**DOI:** 10.12688/f1000research.2-90.v2

**Published:** 2013-04-10

**Authors:** Kenar D Jhaveri, Peter B Schrier, Joseph Mattana

**Affiliations:** 1 Department of Internal Medicine, North Shore University Hospital and Long Island Jewish Medical Centre, Hofstra North Shore LIJ School of Medicine, Great Neck, NY, 11021, USA; 2 Department of Internal Medicine, Winthrop-University Hospital, Mineola, NY, 11501, USA

## Abstract

The most dramatic development in medical decision-making technology has been the advent of the Internet. This has had an impact not only on clinicians, but has also become an important resource for patients who often approach their doctors with medical information they have obtained from the Internet.  Increasingly, medical students, residents and attending physicians have been using the Internet as a tool for diagnosing and treating disease. Internet-based resources that are available take various forms, including informational websites, online journals and textbooks, and social media.  Search engines such as Google have been increasingly used to help in making diagnoses of disease entities. Do these search methods fare better than experienced heuristic methods? In a small study, we examined the comparative role of heuristics versus the 'Google' mode of thinking. Internal medicine residents were asked to “google” key words to come up with a diagnosis. Their results were compared to experienced nephrology faculty and fellows in training using heuristics and no additional help of internet. Overall, with the aid of Google, the novices (internal medicine residents) correctly diagnosed renal diseases less often than the experts (the attendings) but with the same frequency as the intermediates (nephrology fellows).  However, in a subgroup analysis of both common diseases and rare diseases, the novices correctly diagnosed renal diseases less often than the experts but more often than the intermediates in each analysis.  The novices correctly diagnosed renal diseases with the same frequency as nephrology fellows in training.

## Introduction

In medical problem solving and decision-making, experts often use heuristics, or methods of problem solving for which no formula exists, but are instead based on informal methods or experience ^1^. Heuristics help generate accurate decisions in an economical manner for both time and cost. In a sense, expert strategies are immensely adaptive ^1^. While invaluable in helping the experienced clinician arrive at a diagnosis faster, the use of heuristics is associated with biases inherent in efficient decision making and, therefore, can lead to specific patterns of error ^2^. The use of technology employs an algorithmic, rather than a heuristic, approach to medical problem solving and at speeds much greater than human capacity. Various technologies have been experimented with in medicine for years. Past efforts have included computer programs specifically designed to help clinicians make medical decisions and diagnose conditions more efficiently and accurately ^1, 3^. Electronic medical records and information technology have improved access to and ease of use of patient data. Technology does not merely facilitate or augment decision-making, but it reorganizes decision-making practices ^1^.

## Enter “Dr. Google”

The most dramatic development in medical decision-making technology has been the advent of the Internet. Use of social media tools such as Facebook and Twitter allow for sharing of information and getting information at a much faster rate than previously thought. Search engines have slowly emerged as useful tools to acquire data regarding medical knowledge. Clinicians can utilize search engines to help them with decision-making. Search engines, the most popular of which is Google ^3^, allow for the algorithmic surveying of all available information in an attempt to provide the most meaningful and useful information to the end user. It is plausible that the use of search engines could substantially aid the clinician, especially when dealing with diagnostic or therapeutic challenges involving great complexity and multiple variables, but the effectiveness of search engines as an aid to the clinician is incompletely defined, as suggested by a recent study by Krause *et al.* ^4^.

As technology infiltrates everyday medicine, the debate about the appropriate role for information technology within medicine has intensified ^5, 6^. Early on, concern was raised regarding the utility of search engines to direct patients and clinicians to relevant sources ^7^. More recently, there is mounting anecdotal evidence of miraculous or fantastic accounts of patients and physicians-in-training “googling” the answer to a medical question that had experts stumped ^8^. There have been several small studies looking at the ability of doctors at various levels of training and experience to correctly diagnose a disease using Google based on case presentations from the *New England Journal of Medicine (NEJM)*. Falagas *et al.* did a head-to-head comparison of three learners (two medical students and one “trainee doctor”) in which the learners first provided their diagnoses to *NEJM* cases without help, and then repeated the exercise with the help of Google and Pubmed ^9^. While the findings did not reach statistical significance, the study suggested that use of Google and Pubmed may be helpful in generating a differential diagnosis ^9^. Tang and Ng took 26 cases, also from the case records series in the New England Journal of Medicine, and selected 3–5 search terms for each case and entered them into Google ^10^. Using this approach, the Google search provided the correct diagnosis in 58% of the cases ^10^. The conclusions of the studies were essentially the same: Google (and probably other search engines and algorithmic technologies) appears to be a viable clinical tool to aid in physician diagnosis and learning.

## Comparison

Does “googling” a diagnosis replace an experienced physician’s clinical acumen? “Googling” a clinical question may be especially useful in the case of rare or syndromic diseases, but may be less likely to be useful in diagnosing more common diseases. To assess this possibility, we reviewed and analyzed the use of Google as a diagnostic tool in renal diseases and compared it to the experience of fellows and attending staff. A total of 21 members participated in the study (7 novices, 7 intermediate levels- fellows and 7 experts -attendings). We created 103 pairings of common and uncommon renal diseases with keywords related to the features of the disease using a standard renal textbook as a guide ( Appendix 1). The diseases were then categorized as common or rare based upon the consensus of the investigators. This association was not indicated on the worksheets given to the participants. The order of the questions was then randomized and worksheets were made with approximately fifteen keyword pairings per page. Experts (nephrology attendings) and intermediates (nephrology fellows) were given the entire list of keywords (one page at a time) and asked to identify the associated diseases without any aid. Novices (first- and second-year internal medicine residents) were given approximately three pages at random and asked to use Google to identify the renal disease associated with the keywords. The novices were given standardized instructions requiring that they only use the first ten results (first page of results) returned from a Google search. They were then only permitted to use the first page of each of the ten results that appear on the first Google search page. A detailed instruction sheet is attached for reference ( Appendix 2). The residents were instructed to use any or all of the keywords, as they saw fit, and they were allowed to try different iterations of the keywords if their original search did not yield a diagnosis they were satisfied with. The residents were supervised/proctored by one of the investigators; questions were limited to explanations of the rules. The percent of diagnoses correctly identified from the keywords was identified for each test-taking group, and the groups were compared with each other two at a time. The diseases were then categorized as common or rare based upon consensus of the investigators. Worksheets were created with keywords groupings for each disease listed and space provided for a study participant to record the suspected diagnosis. The association of common versus rare was not indicated on the worksheets given to the participants. The participants were asked to complete as many pages as they were willing to complete. All participating experts answered a total of 229 questions. All participating intermediates answered a total of 254 questions. All participating novices answered a total of 230 questions.

The percent of diagnoses correctly identified from the keywords was identified for each test-taking group and the groups were compared with each other two at a time. A t-test was calculated for each pairing; p-values were calculated using Microsoft Excel. A subgroup analysis was also conducted for common diseases and for rare diseases. Table 1 and Table 2 show examples of the common and rare diseases chosen, and the keywords and their associated diseases, respectively.

**Table 1.** Examples of common and rare diseases used in this study.

Common	Rare
Renal tubular acidosis type IV (hyporeninemic hypoaldosteronism)	Syndrome of apparent mineralocorticoid excess (AME)
Acute interstitial nephritis (AIN)	Tuberous sclerosis
Rhabdomyolysis	Pheochromocytoma
Hepatorenal syndrome	Ethylene glycol poisoning
Thin basement membrane disease	Churgh-Strauss syndrome

**Table 2.** Examples of the Keywords used and their associated diseases.

Keyword	Disease
Severe hypertension, Rapidly progressive acute renal failure, rapid skin thickening, high renin	Scleroderma renal crisis
Oliguric acute renal failure, glomerulonephritis, pulmonary hemorrhage, anti-GBM Ab+	Goodpasture’s syndrome
Progressive glomerulonephritis, fever, fatigue, epistaxis, c-ANCA/PR3-ANCA+	Granulomatosis with polyangiitis
Fever, watery diarrhea, acute renal failure, anemia, thrombocytopenia	Hemolytic uremic syndrome (HUS)
Young woman, hypertension, renal artery stenosis on ultrasound	Fibromuscular dysplasia

## Is “Dr. Google” better than experience?

Overall, with the aid of Google, the novices (internal medicine residents) correctly diagnosed renal diseases less often than the experts (nephrology attendings) (72.2% vs. 84.7%, p<0.001), but with the same frequency as the intermediates (nephrology fellows) (72.2% vs. 71.5%, p=0.795). In a subgroup analysis of common diseases, the novices correctly diagnosed renal diseases less often than the experts (76.6% vs. 90.5%, p<0.001) and intermediates (76.6% vs. 82.3%, p=0.031). However, in a subgroup analysis of rare diseases, the novices correctly diagnosed renal diseases less often than the experts (65.2% vs. 76.1%, p=0.014), but more often than the intermediates (65.2% vs. 56.2%, p=0.029). This study is unique, in that it directly compares heuristic and algorithmic problem solving, using the dominant technology of our time: the Internet via Google. It also addresses which types of problems are best solved using the heuristics of an experienced clinician and which problems benefit most from algorithmic problem solving with the aid of a search engine. Limitations of the short research include single-center study, investigator bias and limited number of participants. Residents and fellows are still in the learning process and using a search engine against them can create a certain bias, as they don’t have the experience yet. While this question will require further study, our findings suggest that for uncommon clinical entities, the use of search engine technology may be able to increase the diagnostic performance of a novice to an intermediate level.

## Would you use Google to help diagnose your patient?

Can the computer really 'out think' the doctor in making a diagnosis? A recent editorial in *The New York Times* ^11^ begs this question as well and suggests that in rare diseases, and in many instances, a computer software program would have saved many lives. This might be true for rarely encountered conditions, but perhaps not for common diseases. Rare diseases are often not diagnosed at the first encounter with a physician, and hence the term “rare”. A computer-based query, as used in Google, might help diagnose a rare illness faster, but cannot substitute for the heuristic thinking process of a physician and the matching of patterns facilitated by a physician’s experience. But, in many cases, the internet can reveal rare cases that can lead to unnecessary testing and anxiety for the patient and the physician. Hence, while search engines and diagnostic programs will likely continue to evolve as diagnostic tools, they can aid, but cannot replace the thought processes of the experienced clinician.

## Figures and Tables

**Appendex 1:**
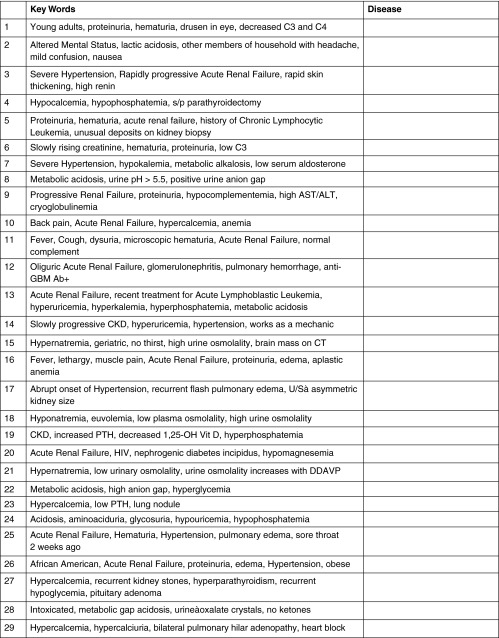
List of key words given to study participants. The first section is the blank keywords and the second is the answer key to the key words. (Section 1, sheet 1).

**Appendex 1:**
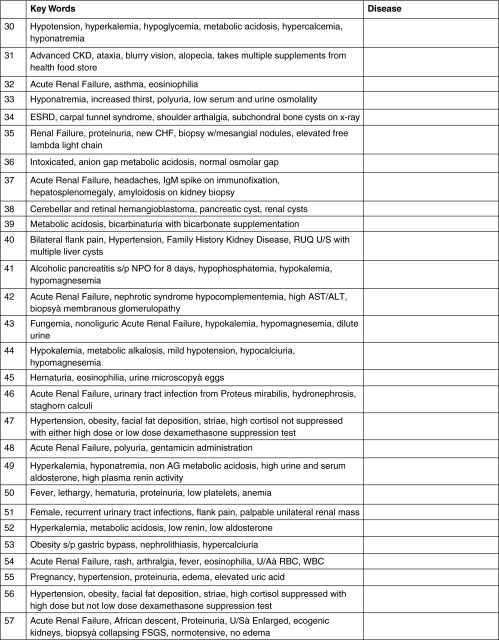
List of key words given to study participants. The first section is the blank keywords and the second is the answer key to the key words. (Section 1, sheet 2).

**Appendex 1:**
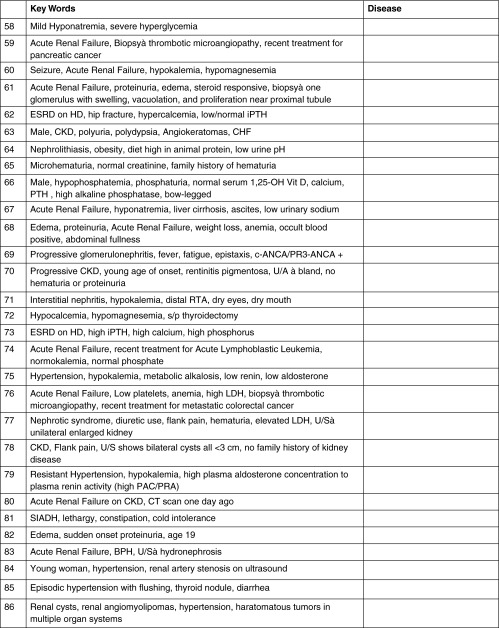
List of key words given to study participants. The first section is the blank keywords and the second is the answer key to the key words. (Section 1, sheet 3).

**Appendex 1:**
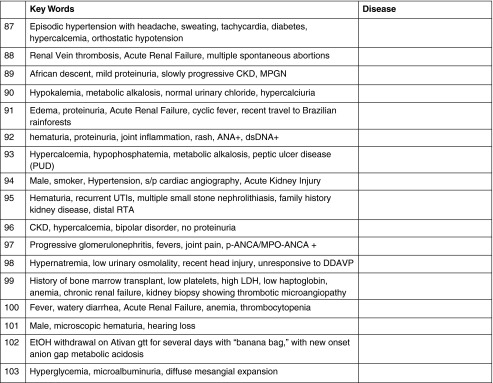
List of key words given to study participants. The first section is the blank keywords and the second is the answer key to the key words. (Section 1, sheet 4).

**Appendex 1:**
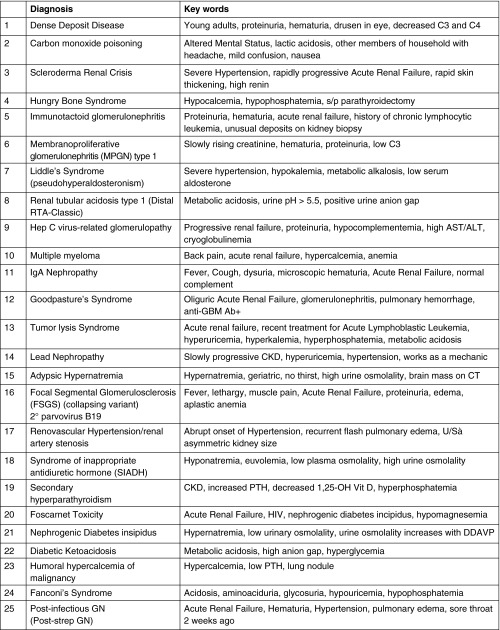
List of key words given to study participants. The first section is the blank keywords and the second is the answer key to the key words. (Section 2, sheet 1).

**Appendex 1:**
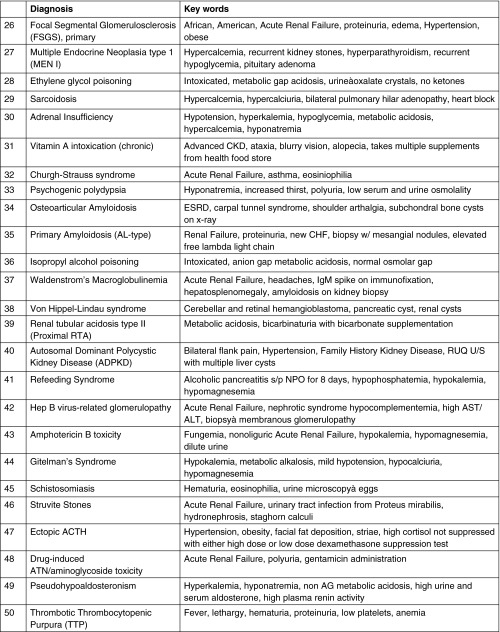
List of key words given to study participants. The first section is the blank keywords and the second is the answer key to the key words. (Section 2, sheet 2).

**Appendex 1:**
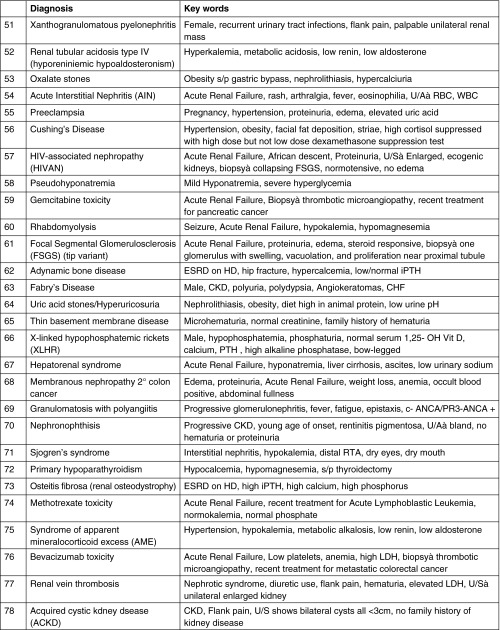
List of key words given to study participants. The first section is the blank keywords and the second is the answer key to the key words. (Section 2, sheet 3).

**Appendex 1:**
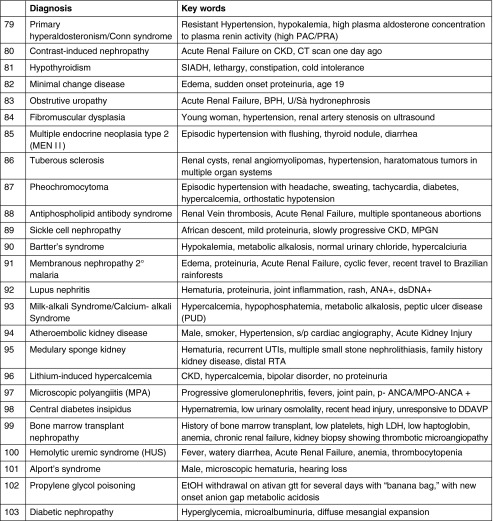
List of key words given to study participants. The first section is the blank keywords and the second is the answer key to the key words. (Section 2, sheet 4).

**Appendex 2:**
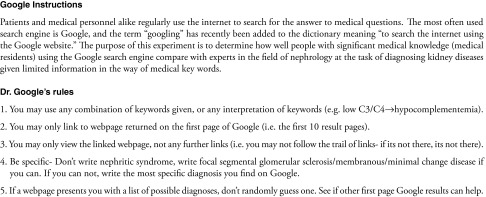
Instruction page given to all “googlers” when asked to participate.
