# Supplementary material for: Paging Doctor Google! Heuristics vs. technology
Source: F1000Res. 2013 Apr 10;2:90. Originally published 2013 Mar 14. [Version 2] doi: 10.12688/f1000research.2-90.v2 (PMC3907161; doi:10.12688/f1000research.2-90.v2)
Supplement: Peer review history

# Peer review: Referee response for version 2

**Published:** 2013-06-03

**Authors:** Rudy Bilous

**Affiliations:** 1 Academic Centre, James Cook University Hospital, Middlesbrough, UK

The addition of an assessment of statistical significance based on the multiple comparisons would improve this paper.

I have read this submission. I believe that I have an appropriate level of expertise to confirm that it is of an acceptable scientific standard.

# Peer review: Referee response for version 1

**Published:** 2013-03-19

**Authors:** Melanie Hoenig

**Affiliations:** 1 Renal Unit, Joslin Diabetes Center, Boston, Boston, MA, USA

I enjoyed the article and was particularly pleased that the authors provided sufficient examples of the keywords used in queries. The commentary was balanced. It is worth noting that while 'Dr. Google' may be helpful in identifying the diagnosis once provided with the keywords or search term, it takes a clinician to be able to sort through the detailed history, physical examination and laboratory data to determine which aspects of the presentation are worth using for a “search.”

I have read this submission. I believe that I have an appropriate level of expertise to confirm that it is of an acceptable scientific standard.

# Peer review: Referee response for version 1

**Published:** 2013-03-19

**Authors:** Rudy Bilous

**Affiliations:** 1 Academic Centre, James Cook University Hospital, Middlesbrough, UK

This is an intriguing report but the approach is a bit simplistic. Atypical presentations of common conditions are more frequently encountered than typical presentations of rare ones. Thus it is really hard to test the hypothesis fully. The internet is more likely to throw up rare options that may result in unnecessary and perhaps dangerous and costly investigations. The fellows are on a journey of understanding and without the experience are likely to score slightly less well than Dr Google. The authors have also made many comparisons and do not appear to have adjusted the level of statistical significance.

I have read this submission. I believe that I have an appropriate level of expertise to confirm that it is of an acceptable scientific standard, however I have significant reservations, as outlined above.
